# A diet based on cured acorn-fed ham with oleic acid content promotes anti-inflammatory gut microbiota and prevents ulcerative colitis in an animal model

**DOI:** 10.1186/s12944-020-01205-x

**Published:** 2020-02-24

**Authors:** J. Fernández, V. García de la Fuente, M. T. Fernández García, J. Gómez Sánchez, B. Isabel Redondo, C. J. Villar, F. Lombó

**Affiliations:** 1 Research Group BIONUC (Biotechnology of Nutraceuticals and Bioactive Compounds), Departamento de Biología Funcional, Área de Microbiología, Universidad de Oviedo, 33006 Oviedo, Principality of Asturias Spain; 2 IUOPA (Instituto Universitario de Oncología del Principado de Asturias), 33006 Oviedo, Principality of Asturias Spain; 3 ISPA (Instituto de Investigación Sanitaria del Principado de Asturias), 33006 Oviedo, Principality of Asturias Spain; 4 Molecular Histopathology Unit in Animal Models for Cancer, Instituto Universitario de Oncología del Principado de Asturias (IUOPA), Universidad de Oviedo, 33006 Oviedo, Spain; 5 Research and Development Department, Cárnicas Joselito S.A., Salamanca, Spain; 6 Department Animal Science, Faculty of Veterinary Medicine, Universidad Complutense de Madrid, Madrid, Spain

**Keywords:** Oleic acid, Acorn-fed ham, Gut microbiota, Ulcerative colitis, Antiiflammatory

## Abstract

**Background:**

Diets based on meat products are not recommended in the case of ulcerative colitis (UC). The objective here is to test if some traditional cured meat products, as acorn-fed ham (high levels of oleic acid), may be useful for controlling inflammatory diseases as UC in animal models, which could represent a new dietary complementary intervention in the prevention of this inflammatory disease in humans.

**Methods:**

Two rat cohorts have been used: conventional vegetable rat feed and acorn-fed ham. UC was induced with DSS in drinking water ad libitum for 1 week. Short-chain fatty acids (SCFAs) and 16S rRNA metagenomics from bacterial populations were analyzed in cecum samples. Colon samples were analyzed for histological parameters.

**Results:**

Acorn-fed ham diet induced changes in gut microbiota composition, with pronounced enrichments in anti-inflammatory bacterial genera (*Alistipes, Blautia, Dorea, Parabacteroides*). The animals with this diet showed a strong reduction in most parameters associated to ulcerative colitis: disease activity index, macroscopic score of colitis, epitelium alteration in colon mucosa, inflammatory cell density in colon, myeloperoxidase titers in colon, proinflammatory cytokines (IL-17, IFN-γ). Also, acorn-fed ham diet animals showed increased total antioxidant activity an oleic acid levels in plasma, as well as higher short-chain fatty acid concentrations in cecum (isobutyric, isovaleric and valeric).

**Conclusions:**

In the acorn-fed ham cohort, as a result of the dietary intake of oleic acid and low intake of omega-6 fatty acids, a strong preventive effect against UC symptoms was observed.

## Background

Some traditional foods present in the Mediterranean diet contain nutraceutical compounds with anti-inflammatory bioactivities which may be useful under certain gastrointestinal conditions. Ulcerative colitis (UC) is the most common form of inflammatory bowel disease (IBD), followed by Crohn’s disease (CD). In the European Union, UC affects 178,000 new individuals each year and about 2.1 million patients in total. Though the etiology of both UC and CD is still unknown, they share an inflammatory basis. In UC, for example, there are higher mucosal levels of pro-inflammatory cytokines such as IL-1β, IL-6, IL-17 or TNFα. UC and CD show a linkage in terms of genetic susceptibility as well, such as the NOD2 and IL23R genes, which are involved in immune response to microbes. Also, IBD patients show alterations in gut microbiota characteristics with respect to the canonical bacterial populations from healthy individuals (dysbiosis). This includes increased *Proteobacteria* (such as *E. coli*) and *Bacteroidetes* (such as *Prevotella* spp., as opposed to *Bacteroides* spp.) rates, and lower *Firmicutes* populations [1–8].

Several environmental factors have been proposed to modulate the onset of UC in children and adults. Lower levels of vitamin D are associated with higher UC incidence. Vaginal delivery and breastfeeding seem to be protective factors against UC, as well as rural lifestyle and exposure to pets. All these factors supposedly increase gut microbiota diversity. However, antibiotic therapy before 5 years of age has been linked to increased UC onset, as it is a factor that diminishes gut microbiota diversity. Smoking, sedentary lifestyle, air pollution, infections by *Salmonella* or *Campylobacter*, or colonization by *Mycobacterium avium* also show a positive correlation with UC development, probably because they trigger inflammatory responses in the gastrointestinal tract [9, 10].

Diet is another important environmental factor linked with UC development and relapses. Dietary fiber from vegetables and fruits in a normal diet shows a protective effect. This is probably due to the gut production of SCFAs by microbiota fermentation of fiber, a type of metabolite with anti-inflammatory effects. The protective effect of dietary prebiotic fiber leads to a reduction of gut inflammatory biomarkers in UC patients, such as fecal calprotectin [11–14]. Processed meat foods (sausages, hamburgers, etc.) are risk factors for UC onset. Conversely, a normal diet high in omega-3 fatty acids (low omega-6/3 ratio) is associated with lower risk of UC [12, 15].

In general, Western diets (high saturated fat and high sugar content, high omega-6/3 ratio, low fiber) have been associated with IBD onset. In contrast, the Mediterranean diet (low saturated fat, low omega-6/3 ratio, high fiber) has been associated with an anti-inflammatory gut status, therefore preventing dysbiosis and IBD [8].

One of the anti-inflammatory actors in the Mediterranean diet is its low omega-6/3 ratio. This diet is high in protective omega-3 fatty acids from vegetables (α-linolenic) and fish (eicosapentaenoic (EPA), docosapentaenoic (DPA) and docosahexaenoic (DHA) acids), and low in omega-6 (linoleic, arachidonic or adrenic acids). The omega-6/3 ratio in traditional diets rich in vegetables and fish is considered to be 1, whereas in some European and North American countries this ratio is around 15. This high value induces a pro-inflammatory status associated with increased incidence of cancer, as well as cardiovascular and inflammatory diseases (such as UC). A low omega-6/3 ratio (such as 2:1) has been shown to attenuate inflammatory mediators production in UC animal models, downregulating pro-inflammatory cell populations such as Th1 (which produces IFN-γ), Th2 (which produces IL-4) and Th17 (which produces IL-17A), CD4^+^ T-helper, and at the same time upregulating Treg cell populations titers (which have anti-inflammatory effects, by modulating T-helper cells) [16]. Another anti-inflammatory actor in the Mediterranean traditional diet is its high oleic acid content. This monounsaturated fatty acid is able to reduce gut pro-inflammatory cytokine levels in animal models for UC generated by the chemical inducer dextran sodium sulfate (DSS) [8, 17, 18].

During the course of this research, a rat animal model for UC was induced with DSS (in drinking water, administered ad libitum for 1 week) and the protective effect of a diet based on traditional acorn-fed Iberian ham was tested, in comparison with rat feed. Acorn-fed Iberian ham is a cured meat product with a low omega-6/3 ratio, traditionally from Southwestern Spain and Portugal. This low omega-6/3 ratio is due to the fact that, in these geographical areas, free-range Iberian pigs fed exclusively on acorns (from green oaks and cork trees) and grass during the months prior to their sacrifice. Acorns are seeds with a low omega-6/3 ratio and high oleic acid content (63%). Consequently, these healthy fatty acids are stored in Iberian pig muscle tissue (as ham) during the free-range feeding months of these pigs on acorns [19]. Continuous use of this traditional cured acorn-fed ham in the human diet is interesting as it provides a gut anti-inflammatory status regarding important gut disorders as UC.

## Methods

### Animals and experimental design

A total of 20 male Fischer 344 rats were maintained in the Animal Facilities at the University of Oviedo (authorized facility No. ES330440003591). All rat experiments were approved by the Ethics Committee of the Principality of Asturias (authorization code PROAE 23/2016).

Rats (5 weeks old) were divided into 2 cohorts of 10 individuals each and fed ad libitum. Rats were maintained in individual cages at controlled temperature, humidity and light cycle. Cohort 1 was fed with universal feed (2014 Teklad Global 14% Protein Rodent Maintenance Harlan diet feed). Cohort 2 was fed only with acorn-fed Iberian commercial ham. Tables 1 and 2 show the nutritional composition and fatty acids composition of the two different diets used in this study. Every day, 25 g of the corresponding ham was added to each rat cage, and the leftovers discharged the next day. The daily ham diet consisted in cubic pieces of 1 cm size, which were stored at 4 °C before daily addition to rat cages. Feed and ham nutritional composition is referred on Tables 1 and 2.

**Table 1:** Nutritional composition of acorn-fed ham and rat feed

	Humidity %	Protein %	fat %	Fiber %	Chlorides %	Ash %	Nitrates ppm	Nitrites ppm
Acorn-fed ham	38.3	31.1	21.4	0	4.50	5	15.04	0.47
Feed	6.9	14.3	4	22.1	0.3	4.7	0	0

**Table 2:** Fatty acids composition of acorn-fed ham and rat feed. One batch per food was measured

Fatty acids		Acorn ham %	Feed %
C14:0	Myristic	1.37	–
C16:0	Palmitic	20.81	14.72
C16:1	Palmitoleic	3.25	–
C17:0	Margaric	0.22	–
C17:1	Heptadecenoic	0.26	–
C18:0	Stearic	7.96	2.94
C18:1	Oleic	51.92	20.58
C18:2n6	Linoleic	10.26	58.82
C18:3n3	α-linolenic	0.75	2.94
C20:0	Arachidic	0.12	–
C20:1n9	Eicosenoic	1.16	–
C20:4n6	Arachidonic	1.30	–
C20:5n3	Eicosapentaenoic	0.08	–
C22:4n6	Adrenic	0.18	–
C22:5n3	DPA	0.17	–
C22:6n3	DHA	0.18	–
		100%	100%
Saturated FA		30.49	17.66
Monounsaturated FA		56.59	20.58
Polyunsaturated FA		14.09	61.76
ω-3		1.18	2.94
ω-6		11.74	58.82
ω-6/ω-3		9.97	20.40

### UC induction and monitoring

One week after the arrival of the animals to the animal facility, the two respective diets started. After one week feeding on the corresponding diet, UC was induced in 8 rats from each cohort. Induction was carried out using autoclaved drinking water containing 3% DSS (40,000 g/Mol, Alpha Aesar) for 7 days, administered ad libitum.

The rats were monitored weekly for food and drinking water intake, weight loss and stool consistency/rectal bleeding using a modified protocol from a published work on UC disease activity index (DAI). DAI is the sum of two parameters: body weight loss (0, more than 5% body weight gain; 1, less than 5% body weight gain and less than 5% body weight loss; 2, from 5 to 10% body weight loss; 3, from 10 to 20%% body weight loss; 4, more than 20% body weight loss); stool consistency (0, normal feces; 1, loose stool; 2, watery diarrhea; 3, slimy diarrhea with little blood; 4, severe watery diarrhea with blood) [7].

### Blood and tissue samples

One week after finishing the administration of DSS, all fasted rats were anesthetized (isoflurane) and sacrificed (pneumothorax) for the extraction of blood (2 mL from heart, centrifuged at 3000 rpm 15 min and then the plasma was frozen), the small intestine (fresh, for Peyer’s patches quantification), the whole colon (fresh or kept in 4% formaldehyde at 4 °C, depending on the test) and the cecum (frozen at − 20 °C). Two rats from each cohort were left free of DSS as absolute controls (no UC).

### Physical measures

The rats were weighed every week during the 3 experimental weeks: at the beginning of DSS administration (day 7), at the end of DSS administration (day 14) and just before sacrifice (day 21).

### Histological studies

#### Colon length

The percentage of its reduction in the experimental samples was calculated with respect to the colons of the 2 control animals from each cohort.

#### Peyer’s patches

Hyperplastic Peyer’s patches were counted along the small intestine. Their number in the experimental animals was calculated with respect to the small intestines’ Peyer’s patches of the 2 absolute control animals from each cohort (animals 9 and 10).

#### Macroscopic score assessment of ulcerative colitis

This parameter was measured by an external investigator, according to a published score. The macroscopic damage score of UC was quantified as: 1, no ulceration and local hyperemia; 2, ulceration without hyperemia; 3, ulceration and inflammation in only one site; 4, two or more ulceration and inflammation sites; 5, ulceration bigger than 2 cm; value 6 to 11, one score point per each 1 cm of extra ulceration [20].

#### Reparative changes in colon mucosa, colon epithelium alterations and inflammatory cell density in colon

The distal colon samples were opened along the longitudinal axis and fixed for 24 h in 4% phosphate-buffered formaldehyde at room temperature before being embedded in paraffin blocks, in accordance with routine procedure. Specimens were sectioned in 5 μm thick sections and were stained using hematoxylin and eosin. Microscopic diagnosis was performed on microphotographs obtained by an Olympus BX-53 microscope and a DP73 digital camera connected to a computer with CellSens software. The images were used to identify widespread epithelial erosions, the degree of loss of goblet cells and crypts, and the degree of inflammatory infiltrate (from mucosa to submucosa), as well as the presence of lymphoid follicles. The colon epithelium alteration score was quantified as: 0, no alteration; 1, focal loss of caliciform cells; 2, extensive loss of caliciform cells; 3, loss of crypts lower than in 50% mucosa surface; 4, loss of crypts in more than 50% mucosa surface and/or polypoid regeneration. The inflammatory cells were analyzed for type (lymphocytes, plasma cells and neutrophils), intensity (mild, moderate and severe degree) and the presence of reparative changes (with or without epithelial regeneration and mucin depletion). The inflammatory cell density in colon mucosa score was defined as: 0, no inflammation; 1, mild inflammation; 2, moderate inflammation; 3, severe inflammation.

### Myeloperoxidase assay in colon mucosa

A 0.5 cm longitudinal section from each colon was excised and this pro-inflammatory enzyme was quantified following a published protocol [21].

### Total antioxidant capacity in blood plasma

Total antioxidant activity was measured in plasma samples using a commercial FRAP (ferric reducing activity of plasma) assay kit (Bioquochem SL, Ref. Kf-01-003). A standard curve of different Trolox (a vitamin E analogue) concentrations was used for comparison.

### Pro- and anti-inflammatory cytokines analysis in blood plasma

IFN-γ, IL-1β, IL-6, IL-10, IL-17a, TGF-β1 and TNF-α tests were performed on blood plasma samples, using commercial Elisa kits (Abnova Ref. KA0273, KA1502, KA0278, KA0274, KA1001, KA0279, KA0280) and following the manufacturer’s instructions.

### GC-MS quantification of SCFAs in feces using deuterated standards

400 mg of frozen cecum feces were thawed and resuspended in 1716 μl milli-Q H_2_0 in 5 ml glass vials, homogenized by vortexing. Then, deuterated SCFAs standards were added as internal controls: deuterated acetate, butyrate, propionate and valerate (Cambridge Isotope Laboratories, USA), to a final concentration of 0.4 mM each. Finally, 400 μl of 50% H_2_SO_4_ and 800 mg NaCl were added. This mixture was resuspended and 1 ml of ethyl acetate was added as an extraction solvent. Samples were stirred for 1 h at 300 rpm and 25 °C, and centrifuged for 5 min at 3500 rpm. 500 μl of supernatants were transferred to a new vial. This extraction was repeated twice.

The GC-MS equipment was an Agilent 7890A (Agilent Technologies) equipped with an inert XL MSD with a triple-Axis detector. Acquisition was done using Chemstation software. The capillary chromatographic column was DB-FFAP (30 m, 0.25 mm ID, 0.25 μm film thickness). Helium was used as the carrier gas at 1 mL/min. Injection was made in splitless mode with an injection volume of 1 μL and an injector temperature of 200 °C. A glass liner with a glass wool plug at the lower end of the liner was used to avoid the contamination of the GC column with nonvolatile fecal material. A blank sample was inserted between experimental samples to check for memory effects.

The column temperature, initially 50 °C (1 min), was increased to 150 °C at 5 °C/min and, finally, to 230 °C at 15 °C/min (total time 20 min). The temperature of the ion source, the quadrupole and the interface were 230 °C, 150 °C and 220 °C, respectively. Scanning ions were 45 and 76 m/z for deuterated propionic acid, 45 and 74 m/z for propionic acid, 43 and 73 m/z for isobutyric acid, 63 and 77 m/z for deuterated butyric acid, 60 and 73 m/z for butyric acid, 60 and 87 m/z for isovaleric acid, 63 and 77 m/z deuterated isovaleric acid, 60 and 73 m/z for valeric acid and 60, 73 and 87 m/z for hexanoic acid. Identification of the SCFAs was based on the retention time of standards and with the assistance of the Wiley 7 library.

### GC-MS quantification of fatty acids in meat samples and blood plasma samples

Lipids from blood plasma samples and *biceps femoris* muscle were extracted and methylated using the procedure described by [22]. Fat extracts were methylated in the presence of sulfuric acid and analyzed by gas chromatography. Previously fatty acid methyl ester (FAME) samples were identified by gas chromatography, as described elsewhere [23]. GC-MS was performed using an HP-6890 (Hewlett Packard, Avondale, PA, USA) gas chromatograph, equipped with a flame ionization detector and capillary column (HP-Innowax, 30 m by 0.32 mm ID and 0.25 μm polyethylene glycol-film thickness). A temperature program of 170 °C to 245 °C was used. The injector and detector were maintained at 250 °C. The carrier gas (helium) flow rate was 2 mL/min. For the identification of each fatty acid, pure standards were used (Sigma). The concentration of individual fatty acids was calculated as a % of total fatty acids. The results were expressed as grams per 100 g of detected FAMEs.

### gDNA extraction and 16S rRNA sequencing for metagenomics

gDNA was extracted from 200 mg of frozen (− 80 °C) cecum feces using E.Z.N.A.® DNA Stool Kit (Omega Bio-Tek Ref. D4015–02), producing 200 μl of genomic DNA. gDNA samples were quantified using a BioPhotometer® (Eppendorf) and their concentrations diluted to 6 ng/μl. These diluted samples were used for performing a PCR amplification following the protocol of Ion 16™ Metagenomics kit (Thermo Fischer Scientific).

PCR amplification products were used to create a library using the Ion Plus Fragment Library kit for AB Library Builder™ System (Cat. No.4477597), with sample indexing using the Ion Xpress™ Barcode Adapters 1–96 kit (Cat. No. 4474517). Template preparation was performed using the ION OneTouch™ 2 System and the ION PGM™ Hi-Q™ OT2 kit (Cat. No. A27739). Metagenomics sequencing was performed using ION PGM™ Hi-Q™ Sequencing kit (Cat. No. A25592) on the ION PGM™ System. The chips used were the ION 314™ v2, 316™ v2 or 318™ v2 Chips (Cat. No. 4482261, 4,483,188, 4,484,355) with various barcoded samples per chip.

### Phylogenetic analysis

The consensus excel table for each metagenomics sequencing was downloaded from ION Reporter 5.6 software. This excel table includes the percentages for each taxonomic level and was used for comparing frequencies between rat individuals and cohorts.

Taxonomic adscription up to species level was performed using the QIIME 2 (v.2017.6.0) open-source bioinformatics pipeline. Analysis of the microbiome community was carried out using R software (v3.2.4): non-supervised multivariate analysis (PCA). For LDA analysis, tab-delimited files were generated in R and computed at family level using Galaxy. Graphical representation of Galaxy output included only discriminative features with logarithmic LDA score higher than 3. The reference library used was the Curated MicroSEQ(R) 16S Reference Library v2013.1; Curated Greengenes v13.5. The number of mapped reads (after the ignored ones due to less than 10 copies) per sample was always over 60.000. Total number of reads was always over 110.000. Counts were normalized by sum scaling. All raw metagenomics data have been deposited at NCBI SRA database (submission PRJNA524796).

### Statistical methods

Data were expressed as the mean value ± S.E.M. Statistical analyses were conducted using Student’s *t*-test when the quantitative data presented normality and the variances were assumed equal. When the variances were assumed different, the Welch’s *t*-test was used. When the quantitative data were not normal, the non-parametric Mann-Whitney U test was used. In the case of qualitative data, the χ^2^ test was used. The graphical representation of all these data was generated using GraphPad Prism software, version 7. In all cases, a *p* value < 0.05 was considered statistically significant (*: *p* < 0.05; **: *p* < 0.005; ***: *p* < 0.0005; ****: *p* < 0.0001).

## Results

### Nutritional composition comparison of acorn-feed ham and feed

Acorn-feed cured ham was analyzed with respect to percentages of humidity, total protein, total fat, total chlorides and total ash. The major difference here was in the total fat content, which was high in acorn-fed ham (21.4%) than in feed (4%) [Table 1].

With respect to the specific composition of fatty acids for acorn-fed ham and rat feed, the main differences between acorn-fed ham and feed are in the oleic acid content, because the content of this monounsaturated fatty acid is much higher in the acorn-fed ham (51.92%) than in the rat feed (20.58%) [Table 2]. Also, the levels of omega-6 fatty acids, considered pro-inflammatory compounds, are 6 times lower in the acorn-feed ham (11.74%) than in rat feed (58.82%). This omega-6 content difference is responsible for a much lower omega-6/omega-3 ratio in acorn-fed ham (9.97) as compared to the feed (20.40) [Table 2].

The nitrate concentration in the acorn-feed ham was 15.04 ppm [Table 1]. This low value is due to the fact that during manufacture of this acorn-feed ham, only sea salt is added, and no chemical preservatives as nitrates nor nitrites are included.

### Effect of acorn-feed ham on body weight and disease activity index

**Fig. 1:**
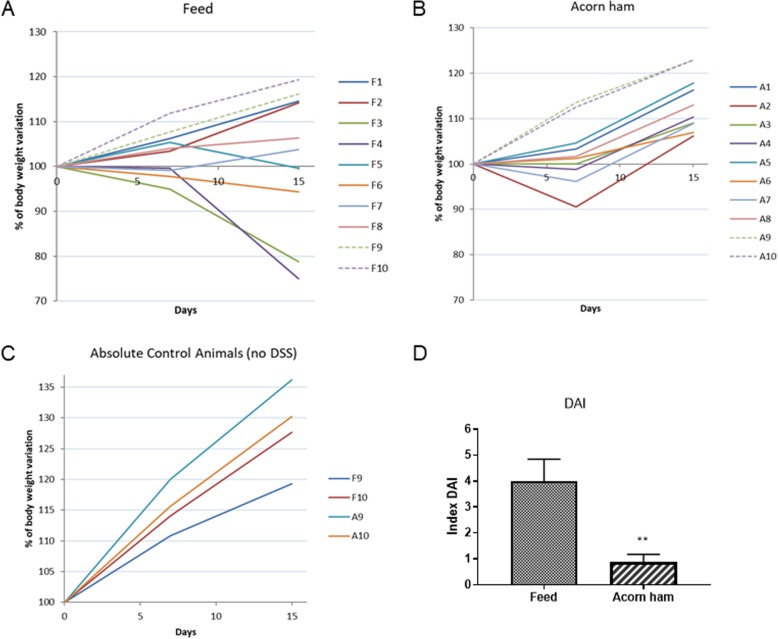
Effect of acorn-fed ham on body weight and disease activity index (DAI). **a**, percentage of body weight reduction in the feed cohort; **b** in the acorn-fed ham cohort; and **c** in the absolute control rats (those lacking the DSS challenge). Data were taken every week during the UC experiment. DSS treatment (UC status) took place between days 7 and 14 of the experiment, and those days are the ones represented on graphics. **d** disease activity index (DAI): this parameter is the sum of two parameters: body weight loss (0, more than 5% body weight gain; 1, less than 5% body weight gain and less than 5% body weight loss; 2, from 5 to 10% body weight loss; 3, from 10 to 20%% body weight loss; 4, more than 20% body weight loss); stool consistency (0, normal feces; 1, loose stool; 2, watery diarrhea; 3, slimy diarrhea with little blood; 4, severe watery diarrhea with blood)

In both cohorts, the animals’ body weight was affected by DSS treatment [Fig. 1]. In the feed cohort, 4 of the UC animals did not recover body weight after finishing the DSS treatment [Fig. 1a]. In fact, these same 4 animals were the ones that later, after sacrifice, showed a higher disease activity index in the colon mucosa (degrees 3 and 4) [Fig. 1d].

In the acorn-fed ham cohort, weight gain slowed slightly during DSS treatment, but this parameter was recovered after the treatment ended. This recovery happened in all 8 animals [Fig. 1b].

The absolute control rats for all cohorts (feed and acorn-fed ham) maintained a continuous and normal weight gain along the experimental weeks and they showed an UC disease activity index of 0, as expected [Fig. 1c].

Finally, disease activity index (DAI) was measured in all animals. DAI values were higher in the feed cohort (4 ± 0.84) than in acorn-fed ham cohort (0.87 ± 0.29) [Fig. 1d].

### Effect of acorn-feed ham on colon histological measurements

**Fig. 2:**
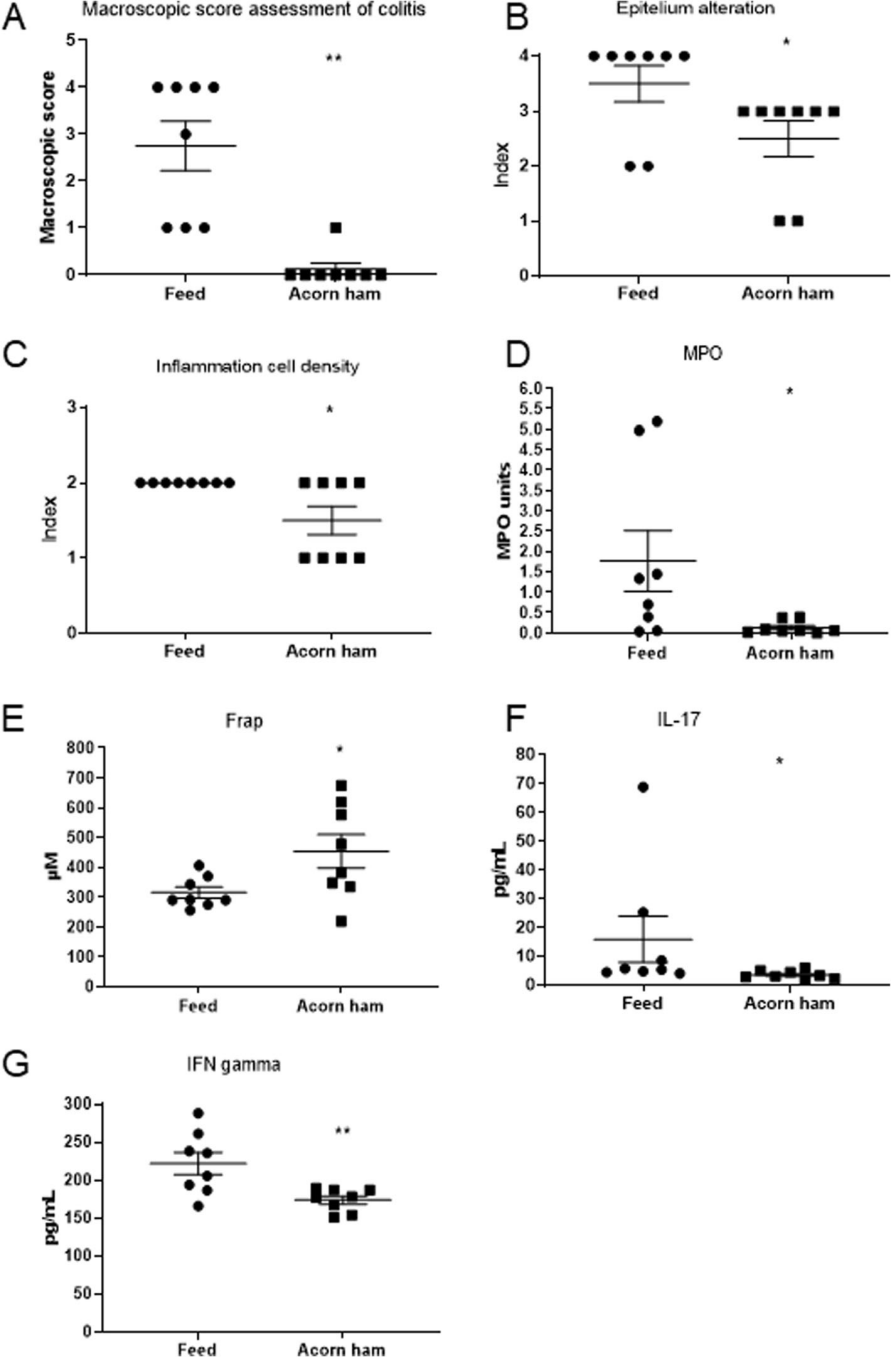
Effect of acorn-fed ham on colon histological measurements, blood plasma total antioxidant capacity and cytokines levels. Circles and squares indicate the corresponding value or score for each rat. **a** macroscopic damage score assessment of UC. **b** colon epithelium alteration score. **c** inflammatory cells density in colon mucosa score. **d** myeloperoxidase assay (MPO). **E**, FRAP total antioxidant capacity. **f** Mean plasma levels of the pro-inflammatory IL-17 cytokine. **g** mean plasma levels of interferon-γ (IFN-γ)

Statistically significant differences were observed between the acorn-fed ham cohort and the feed cohort with respect to the histological measurements assessed. The macroscopic score assessment of UC was much lower (0.12) in the acorn-fed ham cohort than in the feed cohort (2.75), and this difference was statistically significant [Fig. 2a].

The mean score for epithelium alteration in the acorn-fed ham cohort (2.50) was lower than in the feed cohort (3.50) and this difference was statistically significant [Fig. 2b].

**Fig. 3:**
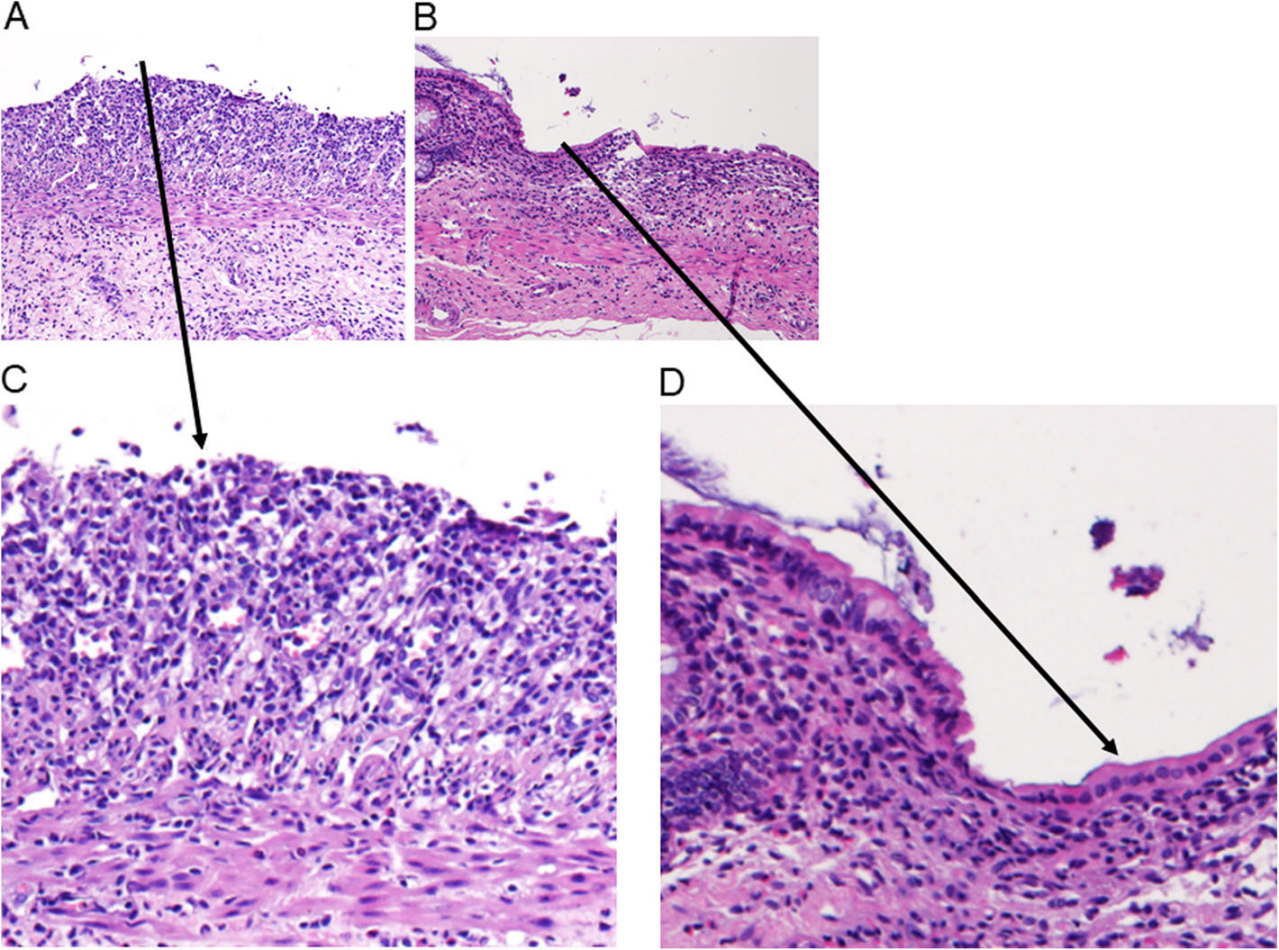
Histology studies on colon mucosa stained with hematoxylin and eosin. **a** Feed cohort, showing moderate inflammation and no re-epithelialization of colon mucosa (10x magnification). **b** Acorn-fed ham cohort, showing mild inflammation and good re-epithelialization of colon mucosa (10x magnification). **c** Feed cohort, showing the no re-epithelialization of colon mucosa at 30x magnification. **d** Acorn-fed ham cohort, showing the good re-epithelialization of colon mucosa at 30x magnification

The mean score for colon mucosa inflammatory cells density in the acorn-fed ham cohort (1.50) was lower than in the feed cohort (2.00) and this difference was statistically significant [Fig. 2c]. Histology studies on colon mucosa revealed that in feed cohort animals, the colon mucosa lacks a structured epithelium monolayer [Figs. 3a and c] due to ulcerative colitis challenge in these animals without re-epithelization. However, in acorn-fed ham animals colon mucosa, it can be easily observed the presence of a proper colon mucosa epithelium structure in a continuous monolayer of cells [Figs. 3b and d].

With respect to the myeloperoxidase assay (MPO), mean myeloperoxidase levels in the colon mucosa from the acorn-fed ham rats were much lower (0.13 MPO units) than in the feed cohort (1.76), and this difference was statistically significant [Fig. 2d].

The three other parameters associated with colon histological studies did not show statistically significant differences between the acorn-fed ham and the feed cohorts. These parameters were the reduction of colon length (which is associated with UC severity) [Fig. S1A], the presence of reparative changes in colon mucosa (which indicates tissue recovery after colon mucosa ulceration) [Fig. S1B] and the number of hyperplastic Peyer’s patches in the small intestine [Fig. S1C]. And finally, the Evans blue assay was also carried out with no statistically significant differences in colon permeability observed between the cohorts [Fig. S1D].

### Effect of acorn-feed ham on blood total antioxidant capacity and cytokine levels on DSS-treated animals

The sacrificed acorn-fed ham cohort rats (1 week after finishing the DSS treatment) showed a much higher total antioxidant capacity (FRAP) in the blood plasma (453.82 μM Trolox equivalent) than the feed cohort rats (315.41 μM Trolox equivalent), and this difference was statistically significant [Fig. 2e].

In terms of cytokines, the main differences observed between the feed cohort rats and the acorn cohort rats were in the levels of IL-17, IFN-γ and TGF-β, though statistically significant differences were obtained only in the cases of the pro-inflammatory IL-17 (3.62 pg/mL mean value in the acorn-fed ham cohort and 15.92 pg/mL mean value in the feed cohort) [Fig. 2f] and IFN-γ (173.81 pg/mL mean value in the acorn-fed ham cohort and 221.96 pg/mL mean value in the feed cohort) [Fig. 2g].

### Effect of acorn-feed ham on short-chain fatty acids concentrations in feces of DSS-treated animals

**Fig. 4:**
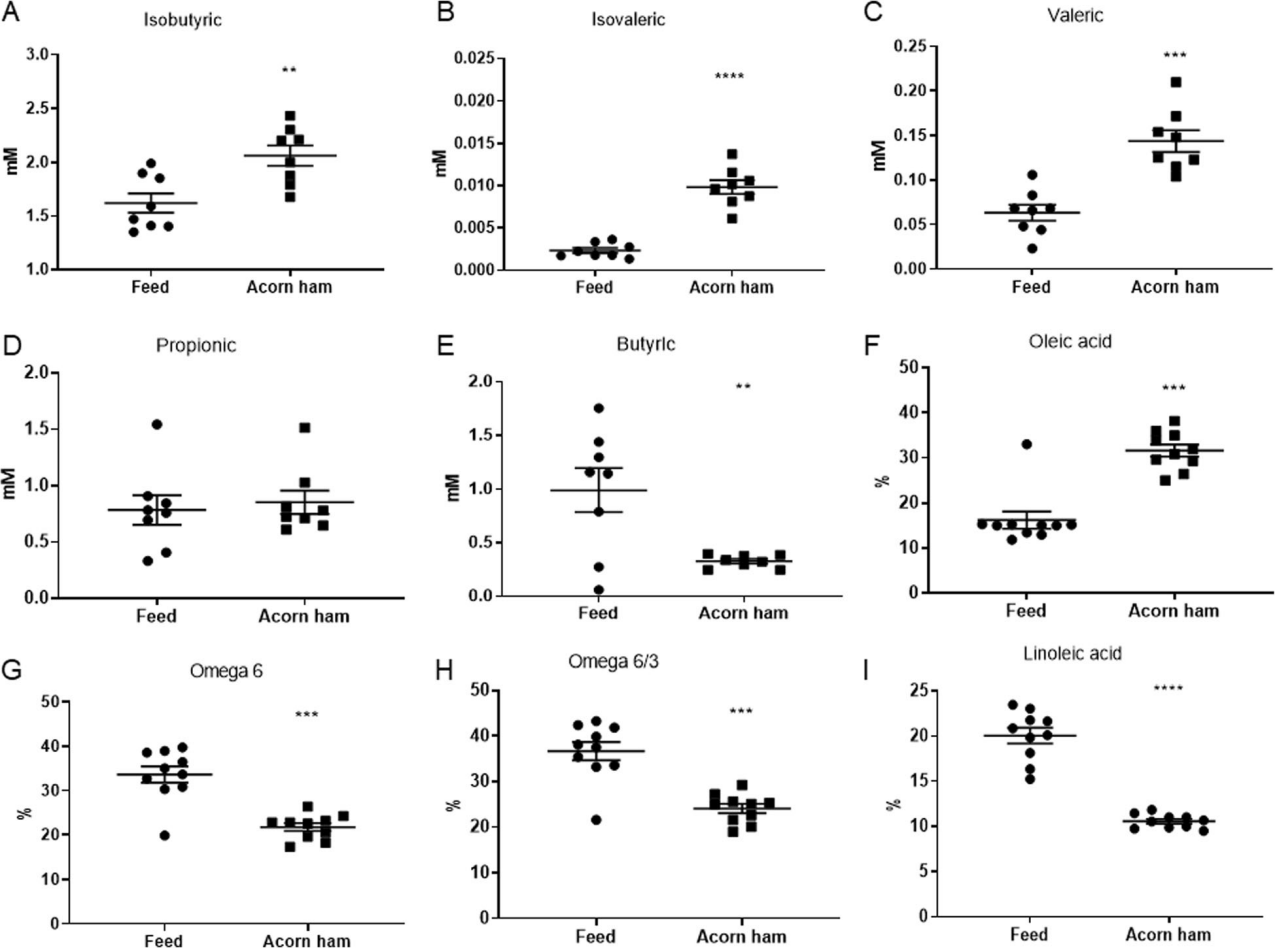
Effect of acorn-fed ham on short-chain fatty acids concentrations in feces and lipids in plasma. **a** isobutyric acid mM concentration in cecum feces. **b** isovaleric acid mM concentration in cecum feces. **c** valeric acid mM concentration in cecum feces. **d** propionic acid mM concentration in cecum feces. **e** butyric acid mM concentration in cecum feces. **f** plasma levels of oleic acid in both rat cohorts. **g** plasma levels of omega-6 fatty acids in both rat cohorts. **h** plasma omega-6/omega-3 ratio in both rat cohorts. **i** plasma linoleic acid in both rat cohorts

Various short-chain fatty acids (SCFAs) were measured by GC-MS in cecum feces collected after sacrifices. These SCFAs were propionic, butyric, isobutyric, valeric and isovaleric acids, which are known compounds involved in colon homeostasis and health. In these quantifications, deuterated standards were used for measurements (see materials and methods section). Statistically significant differences were observed for some of these SCFAs, with higher concentrations in the acorn-fed ham cohort in the cases of isobutyric acid (mean values of 2.06 mM in the acorn-fed ham cohort and 1.62 mM in the feed cohort) [Fig. 4a], isovaleric acid (0.0098 mM in the acorn-fed ham cohort and 0.0023 mM in the feed cohort) [Fig. 4b], and valeric acid (0.14 mM in acorn-fed the ham cohort and 0.063 mM in the feed cohort) [Fig. 4c]. No statistically significant differences were observed with respect to the propionic acid levels between the cohort, although the mean value for propionic acid concentration in cecums from the acorn-fed ham cohort animals (0.8546 mM) was higher than in the feed cohort (0.7851 mM) [Fig. 4d]. Finally, the butyric acid mean value was higher in the feed cohort (0.98 mM) than in the acorn-fed ham cohort (0.32 mM) [Fig. 4e].

### Effect of acorn-feed ham on fatty acids concentrations in blood plasma

Table 3 shows the percentages for each fatty acid in the blood plasmas of the acorn-fed ham cohort rats and feed cohort rats. In accordance with the type of food in each case, animals from the acorn-fed ham cohort showed higher plasma levels of the monounsaturated fatty acid oleic acid (31.61%). This value was double the oleic acid content in the feed cohort rats (16.21%), and the difference was statistically significant [Fig. 4f].

**Table 3:** Mean and SEM fatty acid levels in the blood plasma of rats belonging to acorn-fed ham and feed cohorts

Fatty acids		Plasma levels in acorn-fed ham cohort rats %	Plasma levels in feed cohort rats %
C14:0	Myristic	0.67 ± 0.04	0.88 ± 0.06
C16:0	Palmitic	21.99 ± 0.20	25.59 ± 0.47
C16:1n7	Palmitoleic	1.51 ± 0.11	3.42 ± 0.50
C17:0	Margaric	0.59 ± 0.03	0.77 ± 0.08
C18:0	Stearic	13.40 ± 0.48	11.69 ± 0.66
C18:1	Oleic	31.61 ± 1.33	16.21 ± 1.90
C18:1n-7	11-Octadecenoic	5.15 ± 0.14	3.92 ± 0.22
C18:2n6	Linoleic	10.56 ± 0.24	20.05 ± 0.86
C18:3n3	α-linolenic	0.73 ± 0.05	1.13 ± 0.09
C20:0	Arachidic	0.19 ± 0.02	0.12 ± 0.01
C20:1n9	Eicosenoic	1.02 ± 0.04	0.81 ± 0.12
C20:4n6	Arachidonic	10.03 ± 0.71	12.27 ± 1.05
C22:4n6	Adrenic	1.20 ± 0.06	1.32 ± 0.14
C22:5n3	DPA	0.45 ± 0.02	0.51 ± 0.05
C22:6n3	DHA	1.10 ± 0.07	1.38 ± 0.13
		100%	100%
ω-3		2.28 ± 0.13	3.03 ± 0.18
ω-6		21.79 ± 0.88	33.64 ± 1.85
ω-6/ω-3		24.07 ± 1.01	36.67 ± 2.01

In contrast, the plasma content of the omega-6 fatty acid linoleic acid was much higher in the feed cohort animals (20.05%) than in the acorn-fed ham cohort (10.56%) [Table 3], and this difference was also statistically significant [Fig. 4i]. As this is the main omega-6 fatty acid present in these blood plasmas, this difference resulted in a higher total omega-6 plasma content in the feed cohort animals (33.64%) as compared to the acorn-fed ham cohort (21.79%). It also caused the omega-6/omega-3 ratio in the feed cohort animals to be considerably higher (36.67) than in the acorn-fed ham cohort (24.07) [Table 3]. Both these differences, the total omega-6 content and the omega-6/omega-3 ratio, were statistically significant [Fig. 4g and h].

### Effect of acorn-feed ham on intestinal microbiota

**Fig. 5:**
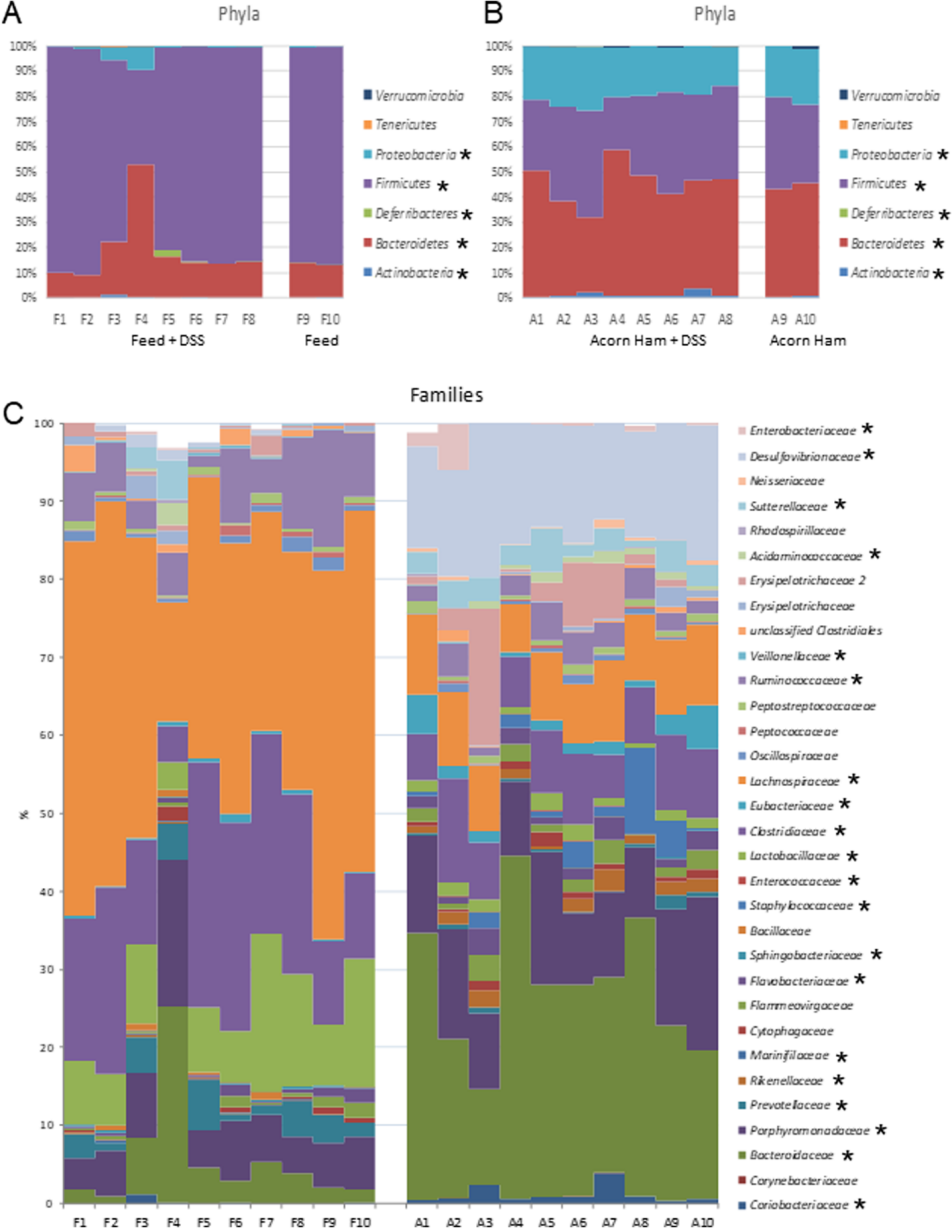
Intestinal microbiota composition (Phyla, Families). Phyla composition (*Verrucomicrobia, Tenericutes, Proteobacteria, Firmicutes, Deferribacteres, Bacteroidetes, Actinobacteria*) for all the surviving animals in this study. **a** feed cohort animals; **b** acorn-fed ham cohort animals. **c** Families composition for all the surviving animals in this study. F: feed, A: acorn-fed ham. An asterisk indicates phyla and families with statistical significant differences between both cohorts

The statistical differences at the phylum level between the two sequenced cohorts is that ham diet showed a increase in *Bacteroidetes* (44.10% versus 17.67% in feed cohort), *Actinobacteria* (1.16% versus 0.15% in feed cohort), and *Proteobacteria* populations (20.41% in ham cohort versus less than 1.81% in feed cohort), and a similar decrease in *Firmicutes* (33.94% in ham cohort versus 79.79% in feed cohort), *Synergistetes* (0% in ham cohort versus 0.11% in feed cohort) and in *Deferribacteres* (0% in ham cohort versus 0.29% in feed cohort) [Fig. 5a, b]. The distribution of these phyla in all the rats treated with DSS was similar to their distribution in the absolute control animals of each cohort, with the exceptions of the F4 rat (and, to a lesser extent, the F3 rat) of the feed cohort, which showed a deep dysbiosis [Fig. 5a].

**Fig. 6:**
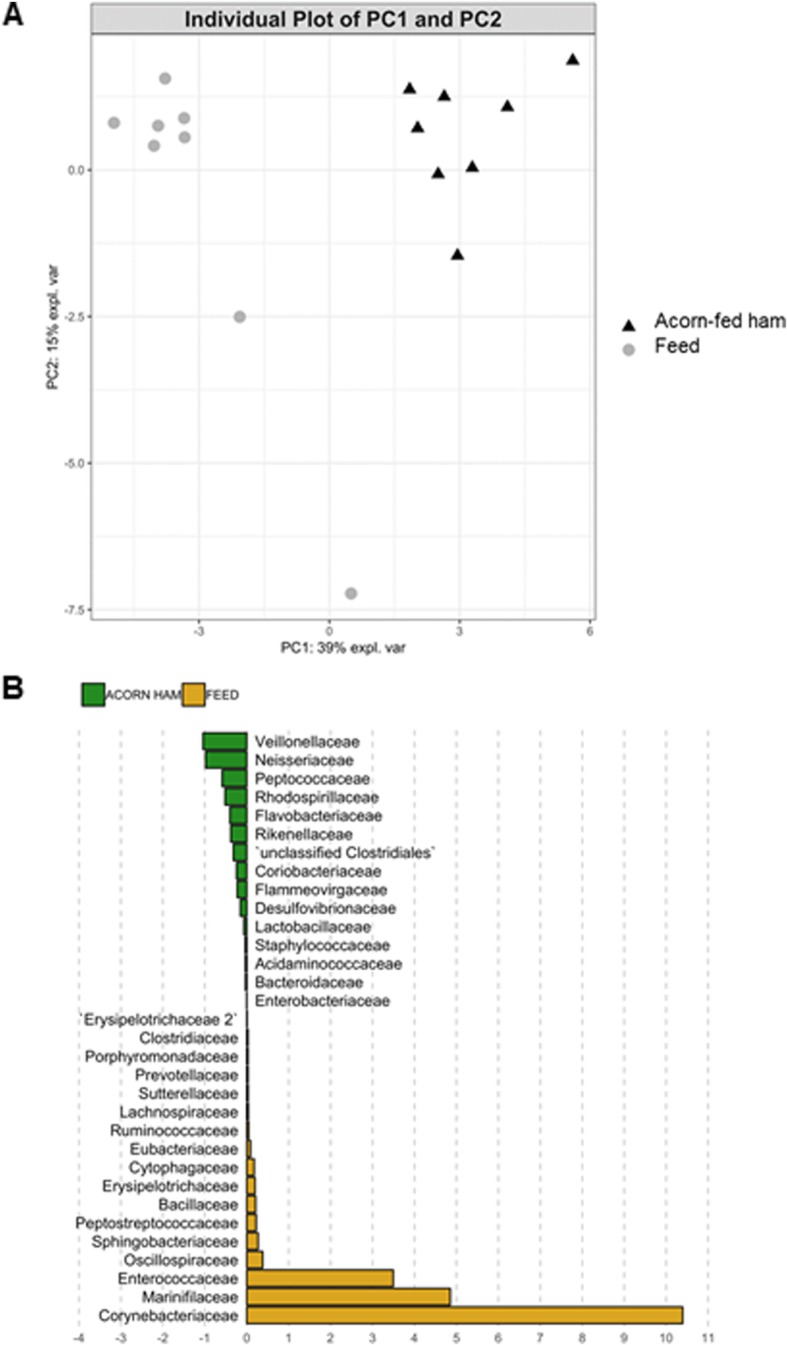
PCA and LDA analyses of gut microbiota composition. A: Gut microbiota PCA cluster analysis, showing that animals belonging to each of the two compared diet cohorts (feed and acorn-fed ham) show very distinctive characteristics. B: LDA analysis showing the families that better discriminate between feed and acorn-fed ham cohorts

At the family level, in general, the composition found in the ham cohort was different from that of the feed cohort animals [Fig. 5c]. The acorn-fed ham cohort animals showed a relatively statistically higher proportion of *Coriobacteriaceae* (*Actinobacteria*, 1.14% in ham cohort in contrast to 0.14% in feed animals)*, Bacteroidaceae* (26.74% in ham cohort in contrast to 5.54% in feed animals), *Porphyromonadaceae* (12.63% in ham cohort in contrast to 7.26% in feed animals) and *Rikenellaceae* (1.51% in ham cohort in contrast to 0.10% in feed animals) (*Bacteroidetes*), *Desulfovibrionaceae* and *Sutterellaceae* (*Proteobacteria*), *Staphylococcaceae* (2.55% in ham cohort in contrast to 0% in feed animals), *Enterococcaceae* (0.04% in ham cohort in contrast to 0.005% in feed animals), *Clostridiaceae Family XIII* (0.13% in ham cohort in contrast to 0.009% in feed animals) *Eubacteriaceae* (2.20% in ham cohort in contrast to 0.42% in feed animals), *Acidaminococcaceae* (0.72% in ham cohort in contrast to 0.3% in feed animals) and *Erysipelotrichaceae* (*Firmicutes*); as well as in *Sutterellaceae* (2.97% in ham cohort in contrast to 0.86% in feed animals), *Desulfovibrionaceae* (15.16% in ham cohort in contrast to 0.56% in feed animals) and *Enterobacteriaceae* (1.55% in ham cohort in contrast to 0.06% in feed animals) (*Proteobacteria*) [Fig. 5c and 6b].

The acorn-fed animals showed a statistically lower proportion of *Marinifilaceae* (0% in ham cohort in contrast to 0.12% in feed animals), *Prevotellaceae* (0.57% in ham cohort in contrast to 3.16% in feed animals), *Sphingobacteriaceae* (0.05% in ham cohort in contrast to 0.18% in feed animals) (*Bacteroidetes*), *Ruminococcaceae* (3.02% in ham cohort in contrast to 7.22% in feed animals)*, Lachnospiraceae* (8.91% in ham cohort in contrast to 37.38% in feed animals), *Clostridiaceae* (8.14% in ham cohort in contrast to 18.87% in feed animals), *Veillonellaceae* (0.02% in ham cohort in contrast to 0.16% in feed animals) and *Lactobacillaceae* (1.41% in ham cohort in contrast to 10.24% in feed animals) (*Firmicutes*); and *Cohaesibacteriaceae* (0.04% in ham cohort in contrast to 0.11% in feed animals) (*Proteobacteria*) [Fig. 5c and 6b].

The animals treated with DSS from each cohort showed a family distribution similar to that of their counterparts without UC induction. The exception, again, was for rats F3 and F4 from the acorn-fed ham cohort [Fig. 5c].

PCA of gut microbiota composition divided the animals in two clusters, indicating differences in the gut microbiota composition associated to both dietary interventions, feed and acorn-fed ham diets (Fig. 6a). Bacterial families with significant differences in their relative abundances between the feed and acorn-fed ham cohorts are indicated in the LDA analysis (Fig. 6b): in total, 32 families explain in a significant way both types of diet.

The main statistically significant differences at the genus level involved a higher proportion in the acorn-fed ham animals of *Bacteroides, Butyricimonas, Parabacteroides, Alistipes, Staphylococcus, Enterococcus, Blautia, Dorea, Absiella, Phascolarctobacterium, Parasutterella* and *Bilophila* [Table 4]*.* On the other hand, the genera *Prevotella, Mucispirillum, Lactobacillus, Clostridium, Lachnoanaerobaculum, Ruminococcus, Oscillibacter* and *Desulfovibrio* showed a reduction in the acorn-fed ham cohort [Table 4]*.*

**Table 4:** Genera and species composition of the cecal microbiota (mean values from all animals in each cohort) in the analyzed animals

Genus	Species	Feed	Acorn ham	Significance
*Adlercreutzia*	*eqolifaciens*	0,000	0,052	***
*Bacteroides*		3973	25,640	****
	*acidifaciens*	0,230	0,021	**
	*caccae*	0,494	1286	*
	*dorei*	0,146	2247	***
	*massiliensis*	0,171	14,970	****
	*uniformis*	0,150	0,469	**
	*vulgatus*	1735	4320	*
*Butyricimonas*		0,070	1071	****
	*virosa*	0,020	0,305	****
*Parabacteroides*		0,981	4808	***
	*distasonis*	0,161	3069	****
	*merdae*	0,709	0,011	****
*Prevotella*		1780	0,012	***
*Alistipes*		0,074	1432	****
	*finegoldii*	0,023	0,812	****
	*indistinctus*	0,002	0,073	***
*Mucispirillum*	*schaedleri*	0,294	0,000	*
*Staphylococcus*		0,000	2485	****
*Enterococcus*		0,000	0,048	****
*Lactobacillus*		10,040	1388	***
	*hominis*	1157	0,044	**
	*intestinalis*	0,345	0,000	**
	*johnsonii*	0,866	0,050	**
	*murinus*	0,666	0,091	**
	*reuteri*	0,571	0,085	**
	*vaginalis*	1001	0,290	**
*Clostridium*		9036	5757	*
*Blautia*		1041	3388	**
	*glucerasea*	0,084	1272	****
*Dorea*		0,002	1045	****
*Lachnoanaerobaculum*	*umeaense*	0,579	0,008	*
*Ruminococcus*		12,670	1911	***
	*gnavus*	6372	1480	**
*Oscillibacter*		0,124	0,072	*
*Absiella*	*dolichum*	0,000	0,424	****
*Phascolarctobacterium*		0,303	0,727	*
	*succinatutens*	0,225	0,530	*
*Parasutterella*		0,868	2831	**
	*excrementihominis*	0,594	2154	**
*Bilophila*		0,134	15,040	****
	*wadsworthia*	0,103	10,430	****
*Desulfovibrio*		0,374	0,000	****

## Discussion

High doses of processed meat products are not recommended in a healthy diet, especially for UC patients. However, some traditional cured meat products, such as acorn-fed ham, contain very high levels of the monounsaturated fatty acid, oleic acid, an anti-inflammatory fatty acid. Furthermore, acorn-fed ham has a lower omega-6/omega-3 ratio than rat feed [Table 2]. The roles of omega 3 and omega 6 fatty acids in DSS-induced UC are not simple and may be influenced by a number of variables. They have been studied extensively, and although in some cases no difference has been found in terms of the protective role of short-term omega 3 dietary fish oil supplementation versus omega 6, in other animal models omega 3 exacerbated the induced UC initially due to a reduction in adiponectin expression in subepithelial myofribroblasts [24, 25]. However, most of the published results point to the anti-inflammatory nature and protective role of the oleic acid and the omega-3 fatty acids against UC. In the case of omega-3 these protective effects are due, in part, to the production of the anti-inflammatory resolvins and reduced titers of TNFα and LTB_4_ leukotrienes [8, 12, 15, 17, 18, 26, 27]. Given the different levels of these fatty acids in the acorn-fed ham compared to other meats, the goal of the study was to test if a diet based on a Mediterranean traditional meat product with a high content of the anti-inflammatory oleic acid and a low omega-6/omega-3 ratio (due to a lower level of the pro-inflammatory polyunsaturated omega-6) would aid in diminishing the UC symptoms in a rat animal model for this disease. Recently, several bioactive peptides generated in cured ham and other fermented meats (*chorizo* sausages) have been associated to beneficial effects, such as antioxidant and cardioprotective ones [28]. The objective here was to demonstrate the benefits of maintaining acorn-fed ham as part of a traditional Mediterranean diet, regarding its protective effects against UC, as an example of a common inflammatory gut condition.

To assess the potential effects of these two diets on UC (control rat feed and experimental acorn-fed ham), once the animals were sacrificed, three histological parameters were studied: macroscopic damage score assessment, colon epithelium alteration and inflammatory cell density in colon mucosa [Fig. 2a, b and c]. In all three cases, statistically significant differences were observed between the acorn-fed ham cohort and the feed cohort. All three parameters indicate the extent of the colon mucosa damage and the pro-inflammatory status and all three were clearly lower in the colons from the acorn-fed ham cohort rats. This indicated either that the acorn-fed ham diet helped prevent damage to the colon mucosa caused by DSS treatment or that the acorn-fed ham diet enhanced the recovery of the affected colon mucosa. A plausible explanation for this is the known anti-inflammatory effect of oleic acid, which is abundant in the acorn-fed ham, as well as its low omega-6/omega-3 ratio [17, 18]. These two parameters support the idea that keeping the traditional acorn-fed ham in a normal diet can provide important anti-inflammatory benefits to the gut health, without the need of drugs nor dietary supplements.

It is also worth noting that the considerable difference in the growth rate of the absolute control animals (those lacking the DSS challenge) in the two cohorts. On average, at the end of the three experimental weeks, the acorn-fed ham cohort rats had a 44% weight gain, while feed cohort rats showed just a 32% weight gain [Fig. 1]. Regarding the feed cohort, animals showed different responses along the UC induction experiment. Rat number F3 and F4, and at a lesser extent F6 and F5, showed a marked reduction in the weight gain, due to a higher damage associated to the UC induction by DSS [Fig. 1a].

Further histological data regarding the myeloperoxidase levels in the colon mucosa also demonstrated this lower pro-inflammatory status in the acorn-fed ham rats. The myeloperoxidase test is usually carried out in UC studies because it serves as a quantitative method for identifying the presence of infiltrated granulocytes in the colon mucosa, a type of immune cell. The higher the myeloperoxidase value, the higher the pro-inflammatory status of the mucosa [29–31]. An analysis of these levels in the two cohorts revealed a statistically significant reduction in the myeloperoxidase levels in the acorn-fed ham rats [Fig. 2d]. In the same way, a statistically significant higher UC disease activity index (DAI) was measured in the feed cohort (DAI level 4) than in the acorn-fed ham cohort (DAI level 0.87) [Fig. 1d].

Along with the histological data, several blood plasma parameters were analyzed in both surviving cohorts. First, the total antioxidant capacity, measured with the FRAP method, was found to be higher in the acorn-fed ham rats, and this difference between the two cohorts was statistically significant [Fig. 2e]. This is most likely due to the higher antioxidant composition (higher levels of monounsaturated and polyunsaturated fatty acids) of acorn-fed ham with respect to feed. Two pro-inflammatory cytokines were also less present in the acorn-fed ham cohort plasma with respect to the feed cohort plasma. These were IL-17 and IFN-γ [Fig. 2f, g]. These immunological parameters present a biochemical explanation for the lesser damage observed in the colon mucosa of the acorn-fed ham cohort animals [Fig. 2].

Similarly, considerable differences were observed in the fatty acid content of the blood plasmas [Table 3]. As expected from a diet rich in oleic acid, the acorn-fed ham cohort animals showed double the amount of oleic acid in their blood plasma [Fig. 4f], lower omega-6 content and a lower omega-6/omega-3 ratio [Fig. 4g and h]. All these parameters indicated a lower pro-inflammatory status in the acorn-fed ham animals, which was also clearly observed at the histological level, as described above.

Other parameters were analyzed in the cecal content, since it is in this organ where fermentation processes are carried out by its microbiota [32]. These analyses allow the identification of metabolic differences associated with the digestion of the three diets in the animals’ cecum [33–35]. Only three of all the SCFAs analyzed were found in a higher concentration in the fecal cecum content of the acorn-fed ham cohort animals: isobutyric acid, isovaleric acid and valeric acid [Fig. 4]. A canonical explanation for the absence of important quantities of butyric acid in the acorn-fed ham cohort animals’ cecum (0.32 mM with respect to 0.98 mM in feed cohort rats) is the fact that ham diets do not supply fiber content [Table 1], the nutrient that is usually fermented by cecum microbiota to generate this SCFA [35, 36].

With respect to gut microbiota changes, the case of the F3 and F4 rats (rat feed cohort) is unique. Although all of the feed cohort animals survived after the DSS challenge, two of them, the F3 rat, and especially the F4 rat, were in critical condition one week after the end of the treatment (DAI score 7 and 8 respectively) [Fig. 1d]. These two rats lost between 21 and 25% of the body weight with respect to week 1 [Fig. 1a]. Also, it is worth noting that the F3 and F4 rats lost this body weight in the week following the withdrawal of DSS from the drinking water [Fig. 1a], i.e., during the expected period of recovery, which indicated a bad prognosis.

The profile of the intestinal microbiota of these two animals (F3 and F4 rats) showed a dramatic alteration at all taxonomic levels examined (especially in the F4 rat) in comparison with the other animals from the feed cohort [Fig. 5a and c]. At the phylum level, the F4 rat showed 52% *Bacteroidetes*, 38% *Firmicutes* and 9% *Proteobacteria* (*Firmicutes*/*Bacteroidetes* ratio of 0.7), while the other rats from the feed cohort showed, on average, 17.6% *Bacteroidetes*, 79.7% *Firmicutes* and 1.8% *Proteobacteria* (*Firmicutes*/*Bacteroidetes* ratio of 4.5). This indicated a deep gut dysbiosis in the F3 and F4 rats with respect to the other animals in the feed cohort. In fact, this phylum distribution for the F3 and F4 rats was very similar to the animals from the acorn-fed ham cohort (44.1% *Bacteroidetes*, 39.9% *Firmicutes* and 20.4% *Proteobacteria*, *Firmicutes*/*Bacteroidetes* ratio of 0.9). This similarity may indicate that the *Firmicutes*/*Bacteroidetes* ratio is probably not sufficient to express the health status of the individual. This is evidenced by the fact that, although both types showed the same *Firmicutes*/*Bacteroidetes* ratio, the F4 rat was in critical condition but the acorn-fed ham cohort animals recovered and thrived.

Nevertheless, even though there were more similarities than differences in the relative proportions of most families present in the F4 rat microbiota as compared to the average values of the acorn-fed ham animals, significant differences were found at the genus and species levels. For example, the relative proportion of the *Parabacteroides* genus was the same, 4.5%, in the F4 rat and 4.8% in the acorn-fed ham cohort animals. But while the distribution at the species level in the acorn-fed ham cohort animals was 3.1% *Parabacteroides distasonis*, 0.7% *P. goldsteinii* and 0.01% *P. merdae*; in the F4 rat these values were 0.7% *P. distasonis*, 0.5% *P. goldsteinii* and 3% *P. merdae*. It is perhaps this distinct distribution at the species level that differentiates a sick animal (such as the F3 and F4 rats) from a healthy one (such as those in acorn-fed ham cohort).

Similarly, the acorn-fed ham cohort animals showed a very different taxa distribution in their intestinal microbiota from feed cohort rats [Fig. 5, 6b and Table 4].

The significance of all these changes is difficult to determine. Likewise, it is difficult to establish the most relevant taxa that were favored with the acorn-fed ham diet, which could be involved in protecting against the DSS challenge. For example, *Bacteroides vulgatus* (*Bacteroidaceae* family) [Table 4] has a relatively high presence in the gut microbiota of the acorn-fed ham cohort animals (4.3%) compared with the feed cohort rats (1.7%). Additionally, several studies have found that it is more commonly present at higher levels in healthy human controls than in UC or IBD patients and it can provide different types of protection against UC [36–41]. However, sialidase activity from *B. vulgatus* mediates the release of sialic acid from intestinal tissue, driving intestinal inflammation and microbial dysbiosis in mice after DSS administration [40]. *B. massiliensis* is present in higher amounts (14.9%) in acorn-fed ham cohort, in comparison with feed cohort (0.1%), a species associated to gut microbiota of healthy subjects [41]. In a similar way, higher levels of *B. dorei* (a species with anti-inflammatory activity) were found in acorn-fed animals (2.2%) in comparison with feed cohort (0.1%) [42].

*P. distasonis* (*Bacteroidetes* phylum, *Porphyromonadaceae* family, [Table 4]) has a greater presence in the acorn-fed ham cohort rats (3.1%) than in the feed cohort rats (0.1%). As in the case of *B. vulgatus*, opposing roles have been assigned to this species in the development of UC: as a reducer of intestinal inflammation in mice treated with DSS by inducing the anti-inflammatory cytokine IL-10 [43], but also as an enhancer of the inflammatory condition in mutant mice affected in the anti-inflammatory intestinal peptidoglycan recognition proteins (Pglyrps) [44]. Perhaps the protective role of *P. distasonis* requires the presence of these Pglyrps proteins in the intestinal mucosa, that is, homologues to these proteins must be present in the wild type animals (*R. norvegicus*) used in this acorn-fed ham cohort and could help *P. distasonis* achieve an anti-inflammatory effect, since animals from the acorn-fed ham cohort with high numbers of this species have a better health status. The UC-associated *Prevotella* genus showed a marked abundance in the feed cohort rats (1.7%) in comparison with the acorn-fed ham animals (0.01%) [45].

As it was indicated in the results section, the acorn-fed ham cohort animals showed a great reduction in phylum *Firmicutes* with respect to the feed cohort animals [Fig. 5a, b]. Two families in this phylum showed the largest reductions in the acorn-fed ham cohort: *Lachnospiraceae* (from 37.3% in feed cohort to 8.9%) and *Ruminococcaceae* (from 7.2% in feed cohort to 3%) [Fig. 5c]. Both families include numerous species with the ability to synthesize anti-inflammatory SCFAs (such as butyrate or propionate) from various polysaccharidic prebiotic fibers [46, 47]. These fibers are present in the feed diet (22.1%) but totally absent in the two types of ham diets. However, other factors apart from the absence of prebiotic fibers in the acorn-fed ham were probably involved in the lower DAI for UC seen in these animals [Fig. 1d]. For example, within *Lachnospiraceae*, the populations of the mucolytic bacteria *Ruminococcus gnavus* were reduced in the acorn-fed ham cohort animals (from 6.4% in feed cohort rats to 1.9%). This bacterium has been reported to be more prevalent and more abundant in CD and IBD patients [48–50] and may play an important role in inducing chronic intestinal inflammation in this study [51].

Nonetheless, even with this decrease in *Lachnospiraceae* populations, some genera increased, such as *Blautia* (1% in the feed cohort rats to 3.4% in the acorn-fed ham animals) and *Dorea* (0.002% in feed cohort to 1% in acorn-fed ham cohort) [Table 4]. UC individuals and CD patients have shown a lower abundance of *Blautia* species than their healthy counterparts [52]. *Blautia* and *Dorea* species could maintain gut homeostasis in terms of its ability to produce the anti-inflammatory SCFA propionate in the acorn-fed ham animals used in this study [47].

With this gut microbiota panorama, the loss of butyrate-synthesizing bacteria that led to a decrease of butyrate in the cecal content [Fig. 4e] could be compensated in the acorn-fed ham cohort rats with an increase in microorganisms able to produce isobutyrate, isovalerate and valerate. An increase in these three SCFAs has been observed by GC-MS of the cecal content of the acorn-fed ham cohort animals [Fig. 4a, b and c].

The higher proportion of *Bacteroidetes* phylum species found in the acorn-fed ham cohort [Fig. 5] could explain the maintenance in SCFAs production. Some of these genera can produce butyrate, such as *Butyricimonas* (0.07% in the feed cohort to 1.1% in the acorn-fed ham animals) [Table 4], [53], but most members of this phylum are mainly propionate producers [47]. *Alistipes* genus (*Rikenellaceae* family) [Fig. 5c and Table 4], for example, was undetectable in the feed cohort animals (0.07%), but accounted for 1.4% total bacteria in the acorn-fed ham cohort. Several studies have linked the presence of *Alistipes* genus with a healthy state [54]. Accordingly, a decrease in this genus has been associated with inflammatory processes [55]. More direct proof of its protective role in the development of UC was observed in the attenuation of DSS-induced UC in mice after gavage with an *Alistipes* strain. In addition to its ability to synthesize SCFAs, succinate is also a significant end product of *Alistipes* metabolism, and this may stimulate SCFAs production by other commensal microorganisms in the gut through the succinate pathway [56]. For example, *Phascolarctobacterium* (*Acidaminococcaceae* family, *Firmicutes*) [Fig. 5c and Table 4] is a succinate-utilizing propionate-producer bacterium with a low presence in the feed cohort rats (0.3%), but accounting for 0.7% in the acorn-fed ham animals [57].

Another notable difference between the gut microbiota of the feed and acorn-fed ham cohorts, not related to SCFAs production, is the presence of the bacteria *Bilophila wadsworthia* (*Desulfovibrionaceae* family, [Fig. 5c and Table 4]). Although this *Proteobacteria* occurs in the intestinal microbiota of healthy humans [58], it has been found to be frequently associated with inflamed appendices in children and adults and it can be considered an opportunistic pathogen [57–60]. In the present study, the presence of *B. wadsworthia* in the feed cohort rats was 0.1%, with the only exception being the dysbiotic F4 rat (1.3%), which was in poor health condition. On the contrary, all animals from acorn-fed ham cohort, treated with DSS or not treated, showed 10.4% *B. wadsworthia* populations. These data are in accordance with a previous work which showed that short-term consumption of a diet based only on animal products changed microbial community structure and increased the abundance of *B. wadsworthia* in the human gut [60].

However, in this study acorn-fed ham animals showing high *B. wadsworthia* populations had a lower DAI than feed cohort rats. In these acorn-fed ham animals, weight gain and recovery were better after the DSS challenge [Fig. 1c]. Also, their pro-inflammatory cytokines plasma levels (such as TNF-α and IL-6) were not statistically different, indicating that more factors than only the presence of *B. wadsworthia* are required for the development of UC in this animal model.

Another proinflammatory *Proteobacteria, Desulfovibrio*, a mucinolytic species (causing a reduction in the mucin barrier, and therefore a lack of protection against pro-inflammatory microbiota) [61], showed a reduction in the acorn-fed ham cohort (0%) in comparison with feed cohort (0.3%). This bacterium is associate to the induction of apoptosis in colon mucosa in in vitro models, and to proinflammatory changes in UC patients [62, 63].

Finally, the *Proteobacteria* of the species *Parasutterella excrementihominis* show a high abundance in acorn-fed ham cohort (2.1%) in comparison with feed diet (0.5%). This species is considered anti-inflammatory, with higher populations present in non-obese individuals [64].

A total of 32 bacterial families explain the main differences in microbiota composition between feed and acorn-fed ham cohorts. *Veillonellaceae, Neisseriaceae Peptococcaceae, Rhodospirillaceae* and other families [Fig. 6b] better describe the effect of acorn-fed diet on gut microbiota composition, whereas *Corynebacteriaceae, Marinifilaceae, Enterococcaceae* and other ones better define the microbiota associated to feed diet [Fig. 6b].

## Conclusions

In conclusion, the acorn-fed ham diet changed the rats gut microbiota due to the different carbohydrate/protein content of the food ingested. The lower carbohydrate and higher protein content in the acorn-fed ham diet led to a decrease in saccharolytic *Firmicutes* species and to an increase in proteolytic *Bacteroidetes* and *Proteobacteria* [Fig. 5]. This dysbiosis caused less butyrate-producing strains, but more isobutyrate, isovalerate and valerate producers, such that total SCFAs amounts in both cohorts were similar, including similar propionate producers [Fig. 4]. Several other beneficial properties from the increased strains in the acorn-fed ham cohort contributed to maintain an appropriate gut homeostasis and to facilitate the recovery of these animals after the DSS challenge. These include some taxons which may secrete metabolites or proteins able to ameliorate inflammation conditions [65], apart from the proven anti-inflammatory effect of oleic acid.

As a second conclusion, the healthy fatty acid composition of the acorn-fed ham, with very high levels of the anti-inflammatory oleic acid and a low omega-6/omega-3 ratio (together with potential bioactive peptides and higher antioxidant activity), may serve as a prevention strategy for UC onset or progression, as it has been demonstrated in this animal model.

The changes observed in the cecal microbiota of acorn-fed ham animals, towards increased populations of anti-inflammatory bacterial species (such as *B. vulgatus, P. distasonis, Parasutterella*), increased populations of SCFA producers (propionate and others) such as *Blautia, Dorea, Phascolarctobacterium* or *Butyricimonas*, and decreased populations of bacteria associated to UC (such as *Prevotella, R. gnavus* or *Desulfovibrio*), together with the anti-inflammatory effect of insaturated fatty acids (specially oleic acid), give rise to the protective effect of acorn-fed ham diet observed in this animal model for UC. Future clinical studies in humans would be necessary to confirm the findings of this UC animal model.

## Supplementary information

**Additional file 1.** Effect of acorn ham on colon and small intestine parameters.

**Additional file 2.** Raw data table.

## Data Availability

All raw metagenomics data have been deposited at NCBI SRA database (submission PRJNA524796). All animals raw analyses data are submitted as supplementary material excel table.

## Abbreviations

CD: Crohn’s disease
DAI: Disease activity index
DHA: Docosahexaenoic acid
DPA: Docosapentaenoic acid
DSS: Dextran sodium sulfate
EPA: Eicosapentaenoic acid
GC-MS: Gas chromatography-Mass Spectrometry
gDNA: Genomic DNA
IBD: Inflammatory bowel disease
ID: Internal diameter
IFN: Interferon
IL: Interleukin
LDA: Linear discriminate analysis
PCA: Principal component analysis
PCR: Polymerase chain reaction
SCFAs: short-chain fatty acids
TGF: Tumor growth factor
TNF: Tumor necrosis factor
UC: Ulcerative colitis
