# Supplementary material for: A diet based on cured acorn-fed ham with oleic acid content promotes anti-inflammatory gut microbiota and prevents ulcerative colitis in an animal model
Source: Lipids Health Dis. 2020 Feb 24;19:28. doi: 10.1186/s12944-020-01205-x (PMC7041278; doi:10.1186/s12944-020-01205-x)

A diet based on cured acorn-fed ham with oleic acid content promotes anti-inflammatory gut microbiota shifts and prevents ulcerative colitis in an animal model

Supplementary Material

J. Fernández,[1] V. García de la Fuente,[4] M. T. Fernández García,[4] J. Gómez Sánchez,[3] B. Isabel Redondo,[2] C. J. Villar,[1] F. Lombó[1*]

[1]Research Group BIONUC (Biotechnology of Nutraceuticals and Bioactive Compounds), Department of Functional Biology, Area of Microbiology, Universidad de Oviedo, Oviedo, Principality of Asturias, Spain. IUOPA (Instituto Universitario de Oncología del Principado de Asturias), ISPA (Instituto de Investigación Sanitaria del Principado de Asturias), Principality of Asturias, Spain.

[2]Department of Animal Science, Faculty of Veterinary Medicine, Universidad Complutense de Madrid, Spain.

[3] Research and Development Department, Cárnicas Joselito S.A., Salamanca, Spain.

[4]Molecular Histopathology Unit in Animal Models for Cancer, Instituto Universitario de Oncología del Principado de Asturias (IUOPA), Universidad de Oviedo.

*Correspondence: F. Lombó, PhD, Biotechnology in Nutraceuticals and Bioactive Compounds (BIONUC) Research Unit, Universdad de Oviedo, Oviedo, 33006, Spain. Tel: +34-985103593, e-mail: lombofelipe@uniovi.es

Figure S1. Effect of acorn ham on colon and small intestine parameters. Circles and squares indicate the corresponding value or score for each rat. **A**, percentage of colon length reduction, in comparison with the mean value for absolute control animals in each cohort. This reduction mean value for acorn-feed ham cohort was lower (14.67%) than in the case of the mean value for feed cohort (20.62%), but this difference was not statistically significant. **B**, presence of reparative changes in colon mucosa: 0, no reparative changes; 1, mild reparative changes (less than 50% of ulcerations surface is re-epithelized); 2, moderate reparative changes (more than 50% of ulcerations are re-epithelized); 3, severe reparative changes (total ulcerations re-epithelization). Although in acorn-feed ham cohort the re-epithelization mean score (1.75) was higher than in feed cohort (1.12), this difference was not statistically significant. **C**, percentage of increase in the number of hyperplastic Peyer's patches in small intestine, in comparison with the mean value for absolute control animals in each cohort. Although in acorn-feed ham cohort (23.53% increase) the mean value of hyperplastic Peyer's patches was lower than in feed cohort (45.31% increase), this difference was not statistically significant. **D**, Evans blue assay showed no statistical significant differences among acorn-feed ham cohort (0.06 µg/mL) and feed cohort (0.10 µg/mL). This assay indicates alterations in colon permeability, which is higher in UC condition.

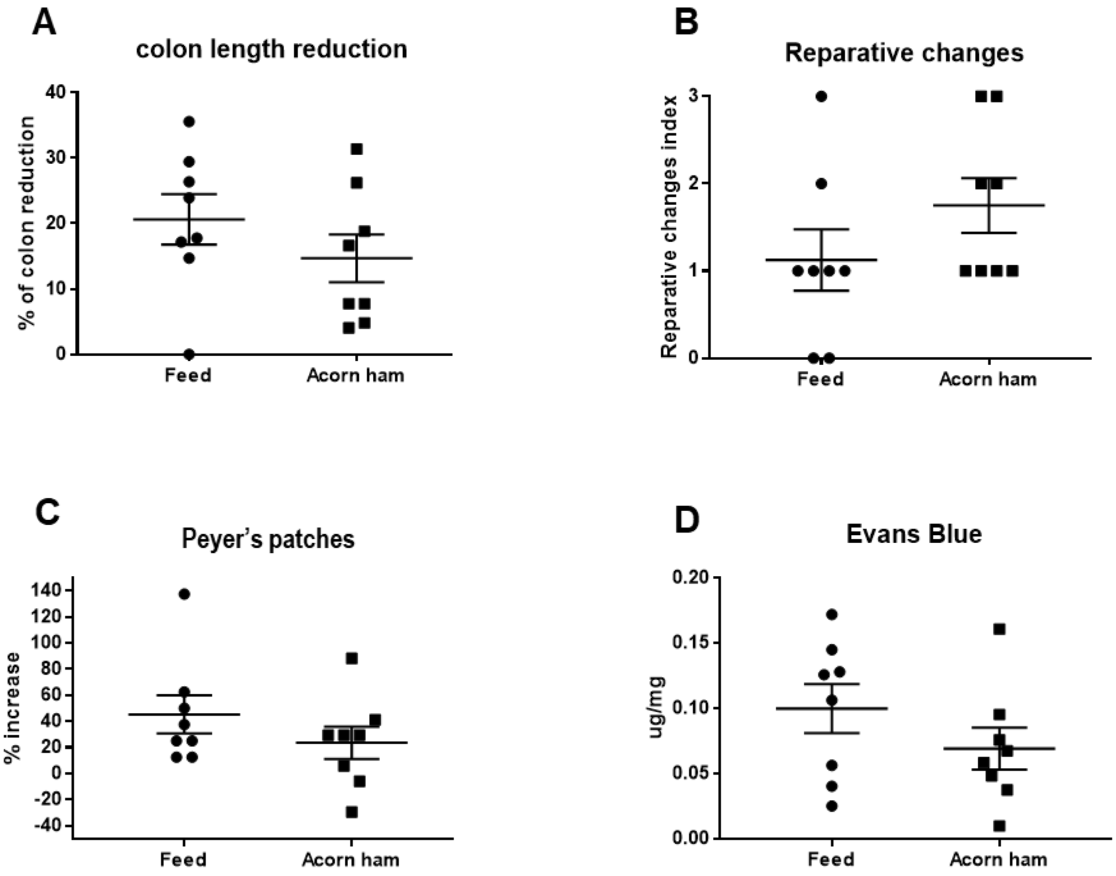

Supplement: Supplementary file 1 — Additional file 1. Effect of acorn ham on colon and small intestine parameters. [file 12944_2020_1205_MOESM1_ESM.pdf]
